# Uncoupling of p97 ATPase activity has a dominant negative effect on protein extraction

**DOI:** 10.1038/s41598-019-46949-4

**Published:** 2019-07-17

**Authors:** Halley B. Rycenga, Kelly B. Wolfe, Elizabeth S. Yeh, David T. Long

**Affiliations:** 1 Department of Biochemistry and Molecular Biology, Medical University of South Carolina, Charleston, SC 29425 USA; 2 Department of Pharmacology and Toxicology, Indiana University School of Medicine, Indianapolis, IN 46202 USA

**Keywords:** Ubiquitylation, Chromatin remodelling, Chaperones

## Abstract

p97 is a highly abundant, homohexameric AAA+ ATPase that performs a variety of essential cellular functions. Characterized as a ubiquitin-selective chaperone, p97 recognizes proteins conjugated to K48-linked polyubiquitin chains and promotes their removal from chromatin and other molecular complexes. Changes in p97 expression or activity are associated with the development of cancer and several related neurodegenerative disorders. Although pathogenic p97 mutations cluster in and around p97’s ATPase domains, mutant proteins display normal or elevated ATPase activity. Here, we show that one of the most common p97 mutations (R155C) retains ATPase activity, but is functionally defective. p97-R155C can be recruited to ubiquitinated substrates on chromatin, but is unable to promote substrate removal. As a result, p97-R155C acts as a dominant negative, blocking protein extraction by a similar mechanism to that observed when p97’s ATPase activity is inhibited or inactivated. However, unlike ATPase-deficient proteins, p97-R155C consumes excess ATP, which can hinder high-energy processes. Together, our results shed new insight into how pathogenic mutations in p97 alter its cellular function, with implications for understanding the etiology and treatment of p97-associated diseases.

## Introduction

p97/Cdc48/VCP (valosin-containing protein) is a highly abundant molecular chaperone that performs a variety of essential functions throughout the cell^1–3^. p97 forms a homo-hexameric AAA+ ATPase that relies on ATP hydrolysis to remodel and disassemble protein complexes^4^. p97 is recruited to substrate proteins by different binding partners that interact with K48-linked polyubiquitin chains^5,6^. K48-linked chains act as the primary signal for p97 recruitment and protein remodeling, although mono-ubiquitin and branched or mixed ubiquitin linkages may play additional roles in regulating p97 activity^7,8^. p97 interacts with multiple components of the 26S proteasome and has been shown to facilitate proteasomal degradation of various proteins^9–11^. However, p97 substrates can also be de-ubiquitinated in the absence of degradation and potentially recycled for future use^12,13^.

In cells, p97 plays an essential role in maintaining protein homeostasis as part of the endoplasmic reticulum-associated degradation (ERAD) pathway^3,14^. Misfolded proteins that accumulate in the endoplasmic reticulum (ER) due to error or stress are recognized by chaperones and directed to ubiquitin ligases that catalyze formation of K48-linked polyubiquitin chains^15^. p97 then facilitates extraction of ubiquitinated proteins from ER or ERAD-associated complexes, followed by protein degradation in the cytoplasm^16^. p97 also plays an analogous role in the nucleus called ubiquitin-dependent extraction from chromatin (UDEC)^17,18^. Extraction and degradation of chromatin-bound proteins is used to regulate dynamic changes in chromatin structure and function during DNA replication, damage signaling, gene expression, and chromosomal segregation^2,12,19–24^.

Due to its central role in many fundamental cellular pathways, disruption of p97 function can lead to several forms of disease. p97 expression is frequently up-regulated in multiple cancers and correlates with poor prognosis in patients^25,26^. These observations suggested that many cancers rely on p97 to manage cellular stress associated with tumorigenesis and that targeting p97 could improve anti-cancer therapeutics^27,28^. A series of p97 inhibitors have been developed that target p97’s ATPase activity, including DBeQ.^29^, ML240^30^, NMS-873^31^, and CB-5083^32^. Although each drug is highly active *in vitro*, CB-5083 has shown the most promise for *in vivo* studies^33^ and use in clinical trials (NCT02223598 and NCT02243917).

Each p97 subunit contains two tandem ATPase domains (D1 and D2) that support inter-subunit interaction and ATP hydrolysis^34,35^. The D1 and D2 domains are flanked by an N-domain and flexible C-terminus that serve as binding sites for different p97 cofactors^5,36^. Although p97 is essential for cellular viability^13,37,38^, heterozygous missense mutations can lead to a collection of related neurodegenerative diseases characterized by multisystem proteinopathy, including frontotemporal dementia (FTD), inclusion body myopathy (IBM), and Paget’s disease of bone (PD)^39,40^. p97 mutations may also contribute to the development of amyotrophic lateral sclerosis (ALS)^41,42^, a progressive neurodegenerative disease affecting the brain and spinal cord.

Pathogenic mutations of p97 are most commonly found within the N-domain, but are also present in and around the D1 and D2 ATPase domains^43^. The majority of these p97 mutations were shown to convey normal or elevated ATPase activity *in vitro*^39,44^, suggesting that many disease symptoms result from elevated or unregulated p97 activity^39,45^. One of the most frequently mutated sites in p97 is arginine 155 (R155), located within the N-domain^40^. Here, we show that a common R155 mutation, R155C, leaves p97’s ATPase activity intact, but functionally uncouples it from protein extraction. As a result, p97-R155C acts as a dominant negative, remaining bound to ubiquitinated substrates and consuming excess ATP. These results reconcile conflicting biochemical and cellular observations, providing new insight on the molecular consequences of p97 dysfunction.

## Results

### Disruption of p97’s ATPase activity prevents its release from chromatin-bound substrates

**Figure 1:**
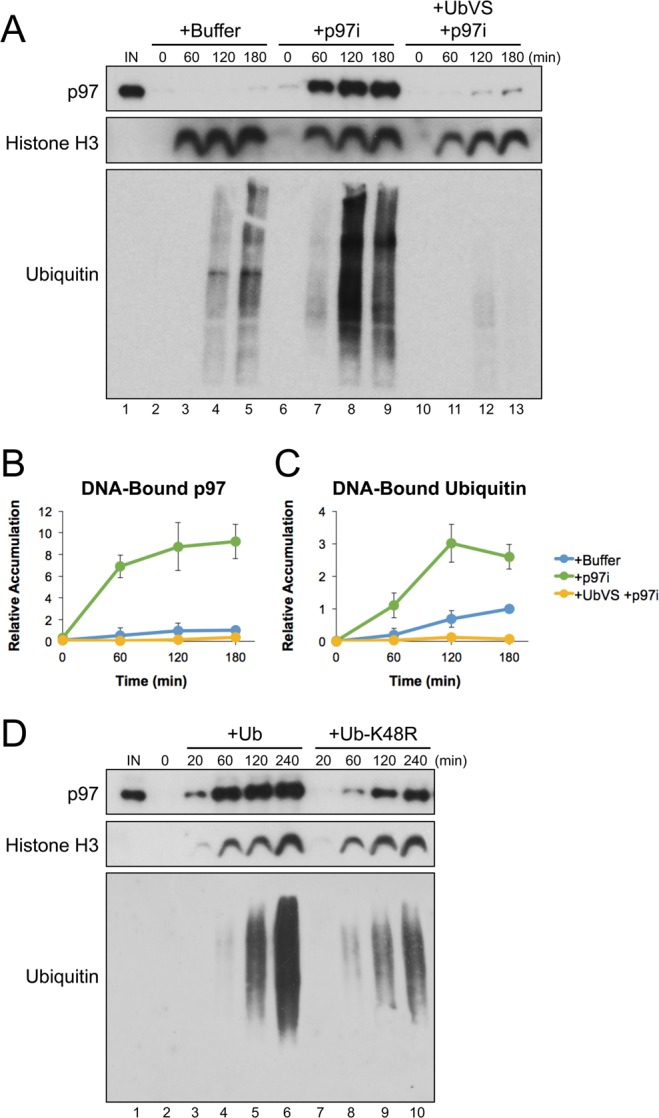
Inhibition of p97 leads to substantial accumulation on chromatin-bound substrates. (**A**) pDNA was incubated in extract supplemented with buffer, the p97 inhibitor CB-5083 (p97i), or p97i and the de-ubiquitinase inhibitor UbVS. DNA-bound proteins were isolated by plasmid pull-down at various times and visualized by Western blot with the indicated antibodies. (**B**,**C**) Relative quantitation of DNA-bound p97 (**B**) and DNA-bound ubiquitin (**C**) from three biological replicates. Values are normalized to peak accumulation in the +Buffer reaction and error bars represent +/− one standard deviation. (**D**) pDNA was incubated in extract supplemented with p97i and either wild-type ubiquitin (Ub) or K48R mutant ubiquitin (Ub-K48R). DNA-bound proteins were isolated by plasmid pull-down at various times and visualized by Western blot with the indicated antibodies. Full Western blot images are available in Fig. S1.

To investigate how p97’s ATPase activity affects its interaction with potential substrates, we analyzed recruitment of p97 to plasmids incubated in *Xenopus* egg extract. When plasmid substrates are added to extract, they are rapidly chromatinized due to an abundance of histones present in egg cytoplasm^46,47^. Chromatinization creates a scaffold for numerous dynamic protein interactions, including those that support chromatin remodeling and DNA compaction^48^. Extract was supplemented with buffer or the p97 inhibitor CB-5083 (p97i). CB-5083 is a highly selective, competitive inhibitor that targets p97’s D2 ATPase domain^32^. Undamaged plasmid DNA (pDNA) was incubated in extract, and then DNA-bound proteins were isolated by plasmid pull-down and visualized by Western blot. Extract treated with buffer showed little or no p97 accumulation on DNA, even after 180 minutes (Fig. 1A, lanes 2–5). However, in the presence of p97i, the level of DNA-bound p97 increased dramatically (Fig. 1A, lanes 6–9). p97 inhibition also led to an increase in ubiquitin on DNA (Fig. 1A, compare lanes 2–5 and 6–9), suggesting that removal of ubiquitinated proteins was defective^13^. Consistent with this inference, we saw that a known p97 substrate, Aurora-B^49^, was enriched on DNA in the presence of p97i (Fig. S1A). We then pre-treated extract with the de-ubiquitinase inhibitor Ubiquitin Vinyl Sulfone (UbVS), which leads to depletion of free ubiquitin^12,50–52^. Loss of ubiquitin prevented the accumulation of both ubiquitinated proteins and p97 on DNA (Fig. 1A, lanes 10–13). Similar results were obtained using the p97 inhibitor NMS-873 (Fig. S2A–C), an allosteric non-competitive inhibitor that also blocks p97’s ATPase activity^31^.

We then asked whether p97 accumulation was dependent on the formation of K48-linked polyubiquitin chains, which act as the primary signal for p97-dependent protein extraction^53,54^. Extract was supplemented with p97i and either wild-type ubiquitin (Ub) or mutant ubiquitin containing an arginine substitution at lysine 48 (Ub-K48R). Compared to reactions supplemented with Ub, Ub-K48R led to a severe reduction in both DNA-bound p97 and ubiquitin (Fig. 1D, compare lanes 3–6 and 7−10). Thus, inhibiting p97’s ATPase activity in extract leads to its accumulation on DNA through interaction with putative substrates modified with K48-linked polyubiquitin chains. Although not all ubiquitinated proteins bound to DNA are likely to be p97 substrates, our results indicate that accumulation of p97 on DNA occurs through its interaction with these proteins.

**Figure 2:**
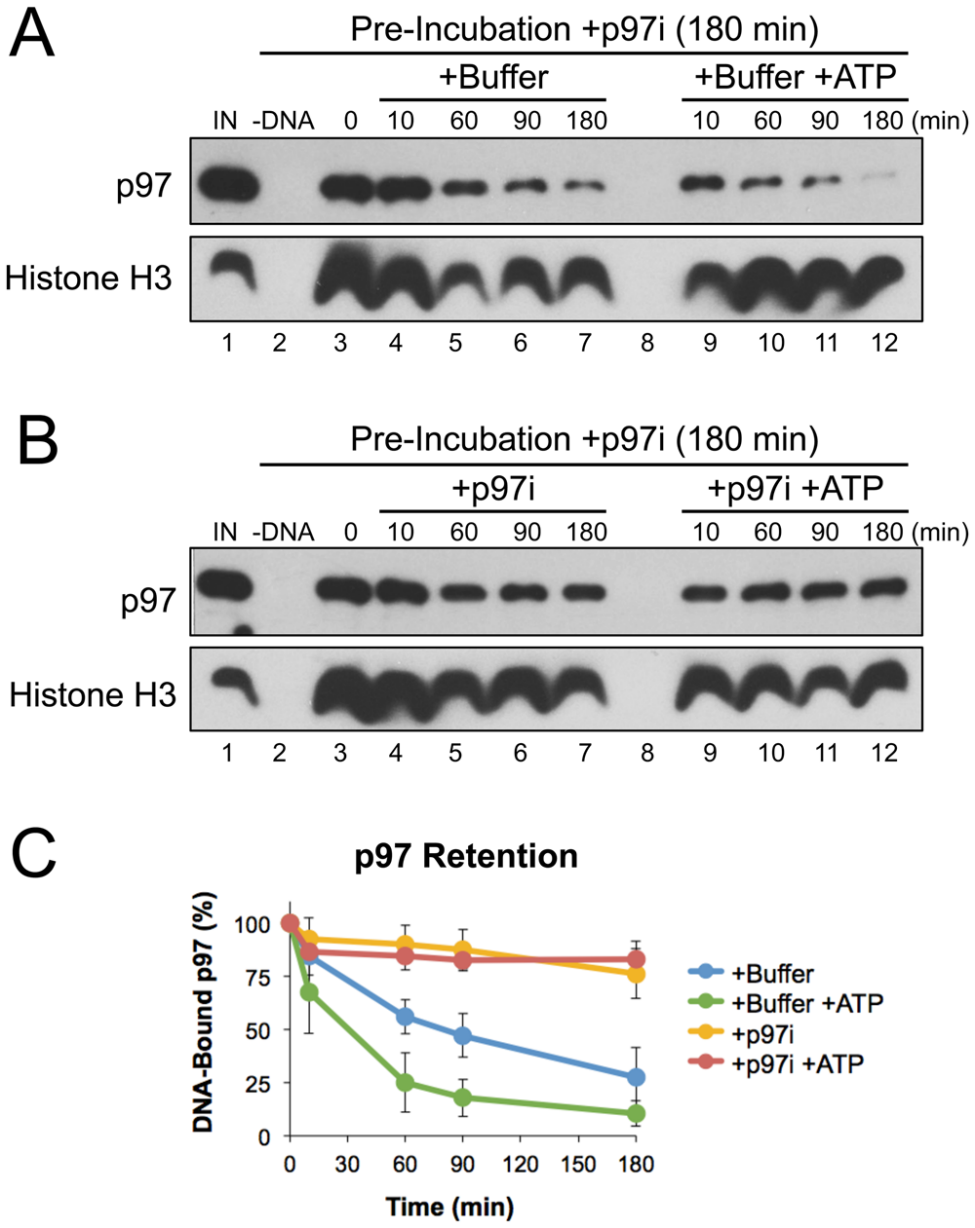
p97 inhibition prevents its release from DNA. (**A**,**B**) pDNA was incubated in extract supplemented with CB-5083 (p97i). After 180 minutes, DNA was isolated by plasmid pull-down and then incubated in buffer +/− ATP (*A*), or buffer containing p97i +/− ATP (**B**). At various times, DNA-bound proteins were recovered and visualized by Western blot with the indicated antibodies. (**C**) Quantitation of DNA-bound p97 from three biological replicates. Values are normalized to accumulation after the 180 minute pre-incubation with p97i (0 minutes, lane 3) and error bars represent +/− one standard deviation. Full Western blot images are available in Fig. S3.

In yeast, ATP hydrolysis was shown to be essential for the release of Cdc48 from ubiquitinated substrates^16^. To test whether inhibiting p97’s ATPase activity also had an effect on the interaction between p97 and its substrates, we first incubated pDNA in extract containing p97i for 180 minutes. DNA was then isolated from extract by plasmid pull-down and suspended in buffer with or without ATP. Retention of p97 on bead-bound DNA was then visualized by Western blot over time. When incubated in the absence of ATP, the level of DNA-bound p97 decreased by ~70% within 180 minutes (Fig. 2A, lanes 4–7; Fig. 2C, blue trace). In the presence of ATP, p97 displacement occurred more quickly, decreasing by ~90% within 180 minutes (Fig. 2A, lanes 9–12; Fig. 2C, green trace). When p97i was included in the incubation buffer, the majority of p97 remained bound at 180 minutes with or without addition of ATP (Fig. 2B, lanes 4–7 and 9–12; Fig. 2C gold and red traces). Using the p97 inhibitor NMS-873, we also saw that inhibition reduced p97’s displacement from DNA in the absence of ATP (Fig. S2D,E). Taken together, these results indicate that loss of ATPase activity influences p97 accumulation in two ways; both by increasing the number of DNA-bound substrates that p97 can interact with and by preventing the release of p97 from those substrates.

### Cellular localization of p97 is revealed by protein inhibition

**Figure 3:**
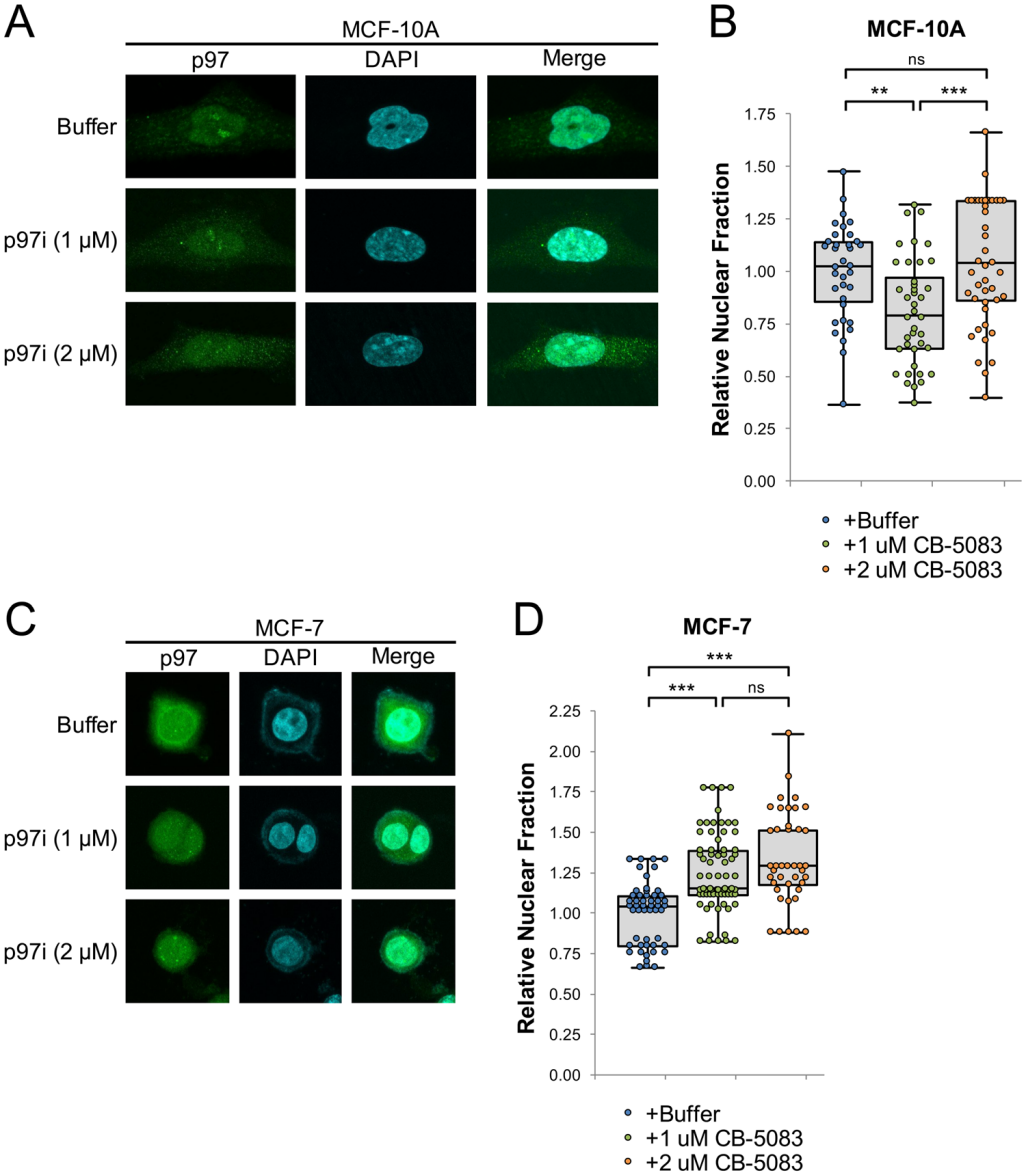
Cellular localization of p97. (**A**) MCF-10A cells were plated for 24 hours and then treated with buffer or the indicated concentrations of CB-5083 (p97i). 24 hours after treatment, cells were visualized by immunofluorescence, with representative images shown (**A**). (**B**) Quantitation of relative nuclear abundance of p97 in (**A**), normalized to the mean in buffer-treated cells. (**C**,**D**) MCF-7 cells were treated, visualized (**C**), and quantitated (**D**), as in (**A**,**B**). Doses above 1.0 μM CB-5083 show elevated levels of the ER-associated stress and apoptosis marker CHOP (Fig. S4C). For each condition, three biological replicates were performed and a total of ≥40 cells were counted. Box plots are drawn from the first to third quartile and the median value is marked by a horizontal line. Whiskers extend to the minimum and maximum values. *p < 0.05, **p < 0.005, ***p < 0.0005, not significant (ns) p > 0.05.

Our *in vitro* results predict that loss of ATPase activity leads to increased accumulation of p97 on chromatin, due to sequestration by ubiquitinated substrates. Although the cellular localization of some p97 mutants has been investigated^55,56^, the specific role that ATPase activity plays in protein localization is not well defined. We hypothesized that p97 inhibition first leads to sequestration of the protein on existing ubiquitinated substrates, prior to substrate accumulation and widespread cellular dysfunction^57,58^. Thus, limited exposure to p97i could provide an indicator of normal p97 utilization within cells. To investigate how p97 inhibition influences its sub-cellular localization in cells subject to different levels of chromatin stress, we treated non-transformed MCF-10A breast epithelial cells and MCF-7 breast adenocarcinoma cells with p97i for 24 hours and then visualized p97 by immunofluorescence. Cells were each treated with a “low” dose of p97i (1 μM), as well as a higher dose (2 μM) that was sufficient to induce expression of CHOP, an apoptosis-associated stress response marker^59^ (Fig. S4A,B). For each treatment, the relative fraction of total p97 that was localized to the nucleus was quantified and normalized to that observed in untreated cells. At the low dose, p97 inhibition had opposite effects on the relative fraction of nuclear p97 in MCF-10A cells, which decreased by 20% (Fig. 3A,B), and MCF-7 cells, which increased by 24% (Fig. 3C,D). These observations support the idea that cancer cells exhibit increased nuclear stress that is alleviated by p97. At the high dose of p97i, the nuclear fraction of p97 increased in MCF-10A cells relative to the low dose and remained elevated in MCF-7 cells, suggesting that induction of apoptosis causes both cell types to retain or re-localize p97 within the nucleus.

**Figure 4:**
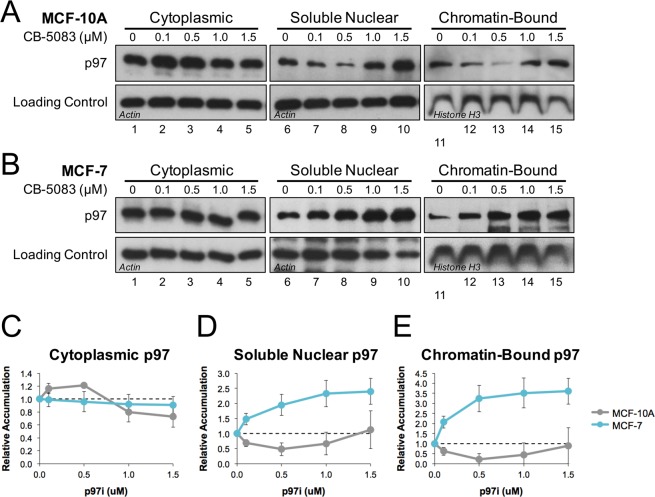
Fractionation reveals p97 accumulation in specific cellular compartments. (**A**) MCF-7 and (**B**) MCF-10A cells were each plated for 24 hours and then treated with increasing amounts of CB-5083 (p97i). 24 hours after treatment, cells were collected and fractionated into cytoplasmic, soluble nuclear, and chromatin-bound fractions. Proteins from each fraction were then visualized by Western blot with the indicated antibodies. Actin and Histone H3 serve as loading controls for the indicated fractions. Full Western blot images are available in Fig. S5. **(C**–**E)** Relative quantitation of p97 accumulation in (**C**) cytoplasmic, (**D**) soluble nuclear, and (**E**) chromatin-bound fractions from three biological replicates. Values are normalized to total p97 levels (Fig. S4) and error bars represent +/− one standard deviation.

To further investigate how p97 is localized within cells, MCF-7 and MCF-10A cells were treated with a range of p97i concentrations, fractionated to isolate different cellular compartments, and then visualized by Western blot. In MCF-10A cells, lower doses of inhibitor led to an increase in cytoplasmic p97 and decreases in both soluble nuclear and chromatin-bound fractions (Fig. 4A,C–E, gray trace). These trends reversed at higher doses, coinciding with induction of CHOP (Fig. S4A). In MCF-7 cells, p97i led to a dose-dependent reduction in cytoplasmic p97, and increases in both the soluble nuclear and chromatin-bound fractions (Fig. 4B,C–E, cyan trace). Taken together, both immunofluorescence (Fig. 3) and fractionation (Fig. 4) data indicate that MCF-10A and MCF-7 cells utilize p97 in different ways, leading to distinct patterns of sub-cellular localization when the protein is sequestered on ubiquitinated substrates.

### The R155C mutation functionally uncouples ATPase activity from protein extraction

**Figure 5:**
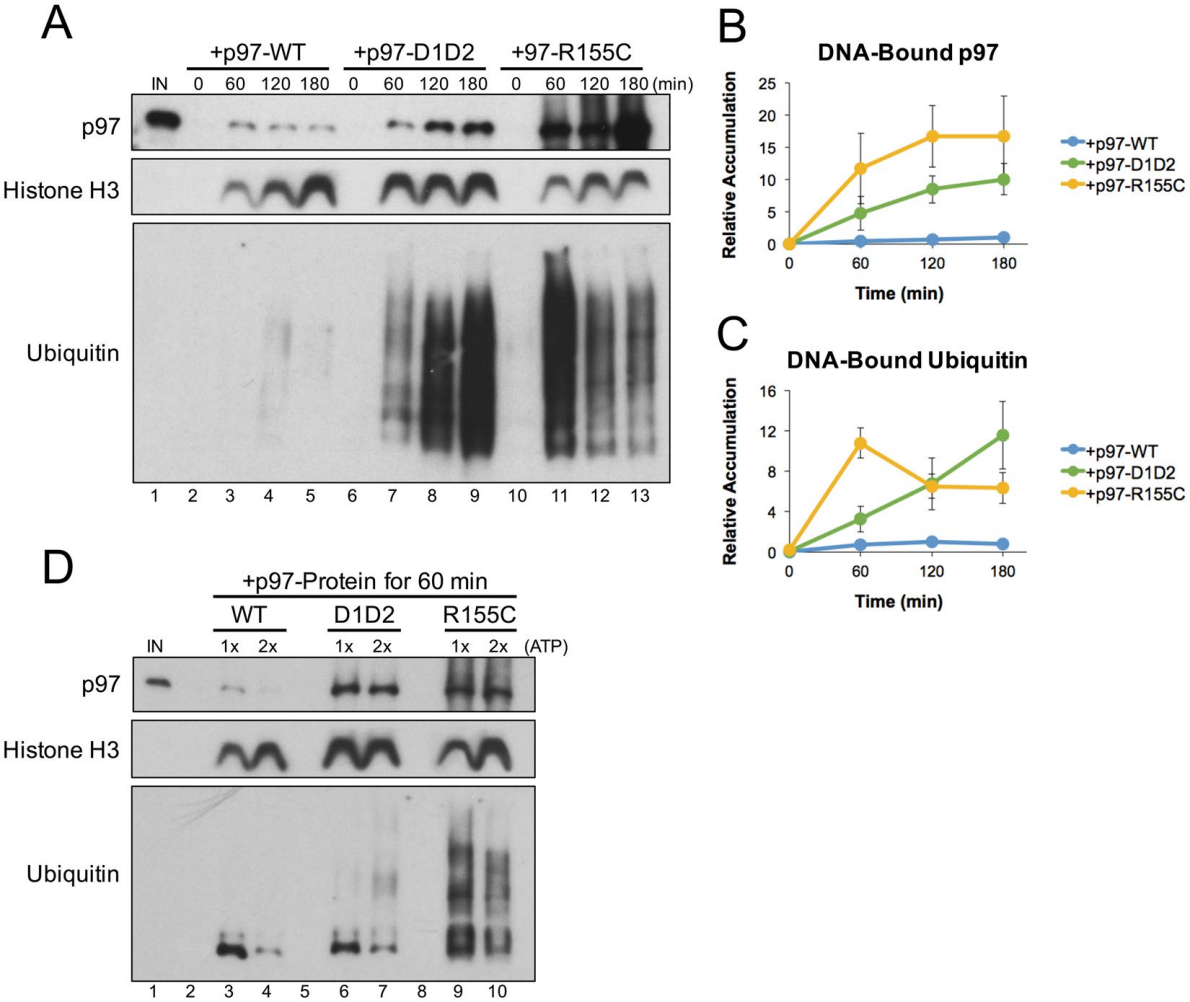
p97-R155C is functionally defective for chromatin extraction. (**A**) pDNA was incubated in extract supplemented with p97-WT, p97-D1D2, or p97-R155C. DNA-bound proteins were isolated by plasmid pull-down at various times and visualized by Western blot with the indicated antibodies. Note that p97 antibodies recognize both endogenous and recombinant proteins. (**B**,**C**) Relative quantitation of total DNA-bound p97 *(***B**) and ubiquitin (**C**) from three biological replicates. Values are normalized to peak accumulation in the +p97-WT reaction and error bars represent +/− one standard deviation. Full Western blot images are available in Fig. S7.

To test how pathogenic mutations in p97 affect its interaction with chromatin-bound substrates, extract was supplemented with recombinant hexameric complexes consisting of wild-type p97 (p97-WT), an ATPase-dead mutant (p97-D1D2)^60^, or p97 containing an R155C mutation (p97-R155C). R155C is one of the most frequently identified p97 mutations and retains ATPase activity *in vitro* (Fig. S6)^44,61^. pDNA was incubated in extract for the time indicated, and then DNA-bound proteins were isolated by plasmid pull-down and visualized by Western blot. Compared to wild-type p97, both the D1D2 and R155C mutants showed an increase in the accumulation of DNA-bound p97 and ubiquitin (Fig. 5A, compare lanes 2–5 with 6–9 and 10–13). Similar defects have also been observed in mouse models carrying different p97-R155 mutations^61–63^. These results indicate that both the D1D2 and R155C p97 proteins have a dominant negative effect on substrate unloading similar to that observed in the presence of p97i (Fig. 1A, lanes 6–9).

Notably, the accumulation of DNA-bound ubiquitin peaked earlier in the presence of p97-R155C (Fig. 5A, lane 11), indicating a defect not observed in the presence of p97i or p97-D1D2. Accumulation of histone H3 on pDNA was also decreased in the presence of p97-R155C (Fig. 5A, compare lanes 2–5 with 6–9 and 10–13). Histone loading is not dependent on p97 (Fig. 1A), suggesting that nucleosome assembly was compromised by an indirect mechanism. We speculated that constitutive activity of p97-R155C’s ATPase domain may consume energy required for normal plasmid chromatinization in extract^64^. When the p97-R155C reaction was supplemented with excess ATP regenerating mix (containing ATP, phosphocreatine, and creatine phosphokinase), the early accumulation of DNA-bound ubiquitin was reduced and histone H3 levels increased (Fig. 5D, compare lanes 9 and 10). Additional ATP also produced a mild increase in histone H3 loading in p97-WT reactions, but had no effect on the early accumulation of ubiquitin or histone H3 in p97-D1D2 reactions (Fig. 5D, compare lanes 1–2 and 4–5). These results indicate that the R155C mutation creates a requirement for additional ATP that can disrupt high-energy consuming processes like chromatinization.

**Figure 6:**
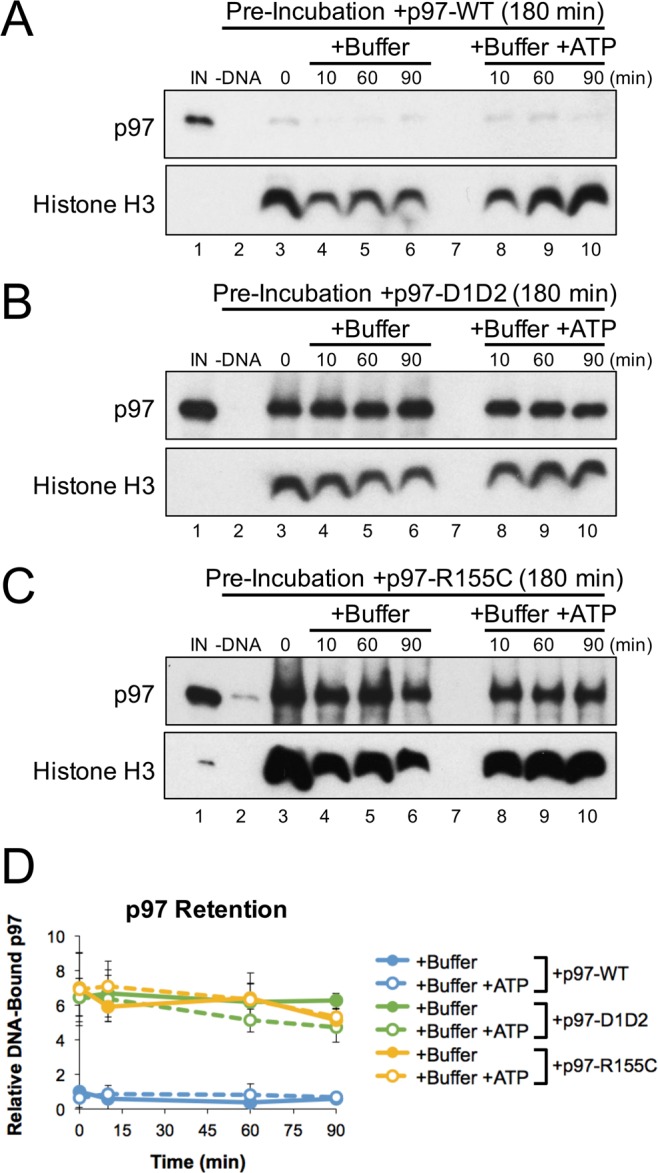
p97-R155C does not utilize ATP for DNA displacement. (**A**–**D**) pDNA was incubated in extract supplemented with p97-WT (**A**), p97-D1D2 (**B**), or p97-R155C (**C**). After 180 minutes, DNA was isolated by plasmid pull-down and then incubated in buffer +/− ATP. At various times, DNA-bound proteins were recovered and visualized by Western blot with the indicated antibodies. (**D**) Quantitation of DNA-bound p97 from three biological replicates. Values are normalized to accumulation after pre-incubation with recombinant p97-WT (0 minutes) and error bars represent +/− one standard deviation. Full Western blot images are available in Fig. S8.

To determine whether the R155C mutation also affects p97’s interaction with DNA-bound substrates, we incubated pDNA in extract supplemented with p97-WT, p97-D1D2, or p97-R155C for 180 minutes. DNA was then isolated from extract by plasmid pull-down and further incubated in buffer with or without ATP. Consistent with Fig. 5A, p97 accumulation was highly elevated in the presence of p97-D1D2 and p97-R155C, but showed little or no accumulation with p97-WT (Fig. 6A–C, lane 3). When pDNA was further incubated in buffer with or without ATP, both p97-D1D2 and p97-R155C remained bound to DNA throughout the reaction (Fig. 6B,C, lanes 4–6 and 8–10). Thus, both mutant proteins were unable to utilize ATP to promote displacement from chromatin. Taken together, Figs 5 and 6 demonstrate that the R155C mutation functionally uncouples p97’s ATPase activity from its protein extraction function.

## Discussion

### ATPase inactivation creates a dominant negative effect

p97 is an essential protein chaperone whose functions are tightly regulated to maintain cellular homeostasis. Using *Xenopus* egg extracts, we found that inhibiting p97’s ATPase activity led to a dramatic increase in the accumulation of both p97 and ubiquitinated proteins on DNA (Fig. 1A, lanes 6–9). Accumulation of p97 was dependent on ubiquitin (Fig. 1A, lanes 10–13) and the formation of K48-linked polyubiquitin chains (Fig. 1D), arguing that it is recruited to DNA through interaction with DNA-bound substrates. Thus, in the context of this system, p97’s normal function is to prevent the accumulation of ubiquitinated proteins on DNA.

Interestingly, the increase in DNA-bound ubiquitin was relatively mild compared to that of p97 (Fig. 1B,C). Using a dilution experiment to monitor p97 binding affinity for its DNA-bound substrates, we saw that p97 inhibition stabilized DNA-bound p97 in the absence of ATP (Fig. 2A,B, lanes 4–7). In yeast, ATP hydrolysis was shown to be essential for the release of Cdc48 from ubiquitinated substrates^16^, suggesting that an induced conformational change helps to disrupt the cofactor-ubiquitin interaction that targets p97 to its substrates. We showed previously that failure to extract a p97 substrate from chromatin can lead to the formation of highly extended polyubiquitin chains^12,29,37^. Together, these data support a model where p97 inhibition stabilizes the binding of multiple p97 complexes to a single, extended ubiquitin chain. As a result, inactive p97 complexes likely shield the substrate from extraction, thereby contributing to a dominant negative effect.

### Localization and utilization of cellular p97

Using a combination of immunofluorescence and cellular fractionation, we compared the cellular distribution of p97 in MCF-10A and MCF-7 cells. When treated with p97i, each cell line showed distinct patterns of p97 localization (Figs 3 and 4). At lower doses, these patterns provide a snapshot of the location and relative abundance of p97 substrates that interact with and sequester inhibited p97. At higher doses, p97 localization is likely dictated by the onset of apoptotic signals (Fig. S4A,B) that promote chromatin decondensation and nuclear collapse^65^. Compared to MCF-10A, MCF-7 cells showed a significant increase in nuclear and chromatin-bound p97 (Figs 3 and 4), suggesting that MCF-7 cells rely more heavily on p97 to manage chromosomal stress associated with rapid cell growth and proliferation. MCF-7 cells are also aneuploid (with the number of chromosomes found in each cell ranging from 60 to 140^66–68^), which could increase the frequency of p97-associated chromatin extraction events generated during cell cycle progression and in response to stress. These results support the idea that p97 represents an attractive target for therapeutic intervention that could provide selectivity during anti-cancer treatments^28,69^.

### Connecting p97 defects with disease etiology

To investigate p97 mutations found in the clinic, we supplemented extract with one of the most common p97 mutants, R155C^40^, and analyzed its effect on chromatin extraction. We found that p97-R155C had a dominant negative effect on the accumulation of DNA-bound ubiquitin and p97 (Fig. 5A, lanes 10–13), indicating a functional defect in chromatin extraction. These results are consistent with previous studies showing that some p97 mutations associated with neurodegenerative diseases also led to an accumulation of ubiquitinated proteins^61–63^. We also observed similar defects when p97’s ATPase activity was inhibited with CB-5083 (Fig. 1A, lanes 6–9) or NMS-873 (Fig. S2, lanes 6–9), and when p97’s D1 and D2 ATPase domains were mutated (Fig. 5A, lanes 6–9). However, by uncoupling rather than disabling p97’s ATPase activity, the R155C mutation also created additional defects (Fig. 5A, lanes 10–13) that were relieved by the addition of excess ATP (Fig. 5D, lanes 7–8). As such, p97-R155C likely binds to ubiquitinated substrates and promotes excessive ATP hydrolysis in a futile attempt to extract the protein. In our system, these defects were observed in the presence of elevated p97 levels and affected what is likely the most ATP-dependent event - chromatinization. However, in cells, excess ATP consumption more likely leads to chronic issues that affect multiple cellular processes. In *Drosophila*, defects associated with several p97 mutations (R155H, R191Q, and A232E) were relieved by increasing ATP levels^45^, suggesting that excess ATP consumption is a common factor in disease etiology. Together, the combination of defects observed with p97 mutations may explain why disease primarily affects high energy-consuming organs and tissues^43^, where the requirement for both p97 function and ATP are highest.

### Treatment of p97-associated disease

Despite p97’s naturally high abundance in cells^70^, many cancers may become increasingly reliant on p97 function to manage elevated stress associated with tumorigenesis^69,71^. Indeed, targeting p97 has been shown to induce apoptosis in multiple cancer cell lines and solid tumor mouse models^27,32,33,72^. p97 inhibitors also show promise for treatment of multiple myeloma, either as a single agent or in combination with proteasomal inhibitors^72,73^. For neurodegenerative diseases associated with p97 mutation, inhibiting ATP hydrolysis could influence p97 activity in different ways. For example, in this report, we show that R155C uncouples ATP hydrolysis from chromatin extraction. Although p97 inhibition could relieve defects associated with excess ATP consumption, it may also exacerbate the dominant negative effects of ATPase inactivation. However, p97 inhibition was recently shown to suppress several mitochondrial defects associated with p97 mutations R155H and A232E, suggesting a balance can be reached that provides therapeutic value^74^. Collectively, our results clarify the consequences of one of the most common p97 mutations and describe how protein dysfunction may contribute to disease etiology. Understanding the role that p97 plays in enabling or preventing different diseases will provide a rationale for targeting specific mechanisms during treatment.

## Methods

### DNA incubation in *Xenopus* egg extracts

*Xenopus* egg extracts were prepared as described previously^75^. Plasmid DNA was incubated in nucleoplasmic extract (NPE) at a final concentration of 50 ng/μL at 21 °C. Plasmids used in this study do not contain DNA damage and are not replicated during the reaction due to the absence of licensing in a high-speed supernatant (HSS) extract^46^. Where indicated, reactions were supplemented with: 150 mM NMS-873 (ApexBio Technology), 500 mM CB-5083 (PharmaBlock Sciences), 150 μM Ubiquitin Vinyl Sulfone (Boston Biochem), 50 μM recombinant ubiquitin (Boston Biochem), or 40 ng/μL recombinant p97. All experiments were performed three or more times and representative results are shown. Experiments involving vertebrate animals (*Xenopus laevis*) were performed in accordance with all guidelines and regulations using an approved protocol administered by the Medical University of South Carolina Institutional Animal Care and Use Committee (IACUC).

### Plasmid pull-down

Isolation of plasmids from extract was performed as described previously^12^. Briefly, streptavidin-coupled magnetic beads (Dynabeads M-280, Invitrogen) were washed three times with Tris Buffer (50 mM Tris pH 7.5, 150 mM NaCl, 1 mM EDTA pH 8, 0.02% Tween-20). Biotinylated LacI was then added to beads (12 pmol of LacI per 6 μL beads) and rotated at 21 °C for 40 minutes. The beads were washed four times with Pull-Down Buffer (10 mM Hepes pH 7.7, 50 mM KCl, 2.5 mM MgCl2, 250 mM sucrose, 0.25 mg/ml BSA, 0.02% Tween-20) and then stored at 4 °C until use. At the indicated time points, reaction samples were added to LacI-coupled magnetic beads and rotated for 20 minutes at 4 °C. Beads were then washed three times with 250 μL of Wash Buffer (10 mM HEPES pH 2.7, 50 mM KCl, 2.5 mM MgCl2, 0.25 mg/ml BSA, and 0.03% Tween 20), dried, and resuspended in 2× SDS Sample Buffer (100 mM Tris-HCl pH 6.8, 4% SDS, 0.2% bromophenol blue, 20% glycerol, and 200 mM β-mercaptoethanol). Plasmid-bound proteins were resolved by SDS-polyacrylamide gel electrophoresis (PAGE) and visualized by Western blotting with the indicated antibodies.

For dilution experiments, plasmids were incubated in extract for 180 minutes, isolated by plasmid pull-down, washed three times with Lac I wash buffer, and then resuspended in 100 volumes of Egg Lysis Buffer (ELB: 10 mM HEPES-KOH pH 7.7, 2.5 mM MgCl2, 50 mM KCl, 250 mM sucrose, and 1 mM DTT) supplemented with 5 mM ATP and/or 500 mM CB-5083 as indicated. After dilution, reactions were incubated at 21 °C for the indicated time, dried, and resuspended in 2xSDS Sample Buffer for analysis by Western blot. The following antibodies were used in this study: p97 (#612182) from BD Biosciences, histone H3 (#PA5-16183) from Thermo Fisher, ubiquitin (#PA1-187) from Pierce Biotechnology, actin (ab1801) from Abcam, and Aurora-B (A300-431AT) from Bethyl Laboratories.

### Protein expression and purification

Expression constructs for *Xenopus laevis* wild-type His6**-**p97 and the D1D2 ATPase-inactive mutant (E305Q and E578Q) were provided by Olaf Stemmann (University of Bayreuth)^76^. The R155C mutation was introduced by Quikchange site-directed mutagenesis (Agilent Technologies) using the following primers:

Forward 5′-GGTGACATTTTCCTGGTACTTGGAGGGATGAGAGCC-3′

Reverse 5′-GGCTCTCATCCCTCCAAGTACCAGGAAAATGTCACC-3′

Recombinant proteins were purified as described previously^76,77^. Briefly, bacterially expressed proteins were bound to Ni-NTA-agarose **(**Life Technologies**)**, eluted with NTA Elution Buffer (1 mM MgCl2, 100 mM KCl, 150 mM sucrose, 5 mM HEPES-KOH pH 7.4, 250 mM imidazole, 1 mM DTT), dialyzed against ELB, and then snap frozen for storage at −80 °C.

### ATPase activity assay

The ATPase activity of recombinant p97 was measured using the ADP-Glo® Luminescent Kinase Assay (Promega). 500 μg of recombinant p97 (WT, D1D2, or R155C) was incubated in 25 μL of ELB containing 0.1 mM ATP with or without 0.1 mM CB-5083 at 21 °C. At the indicated times, 5 μL from each reaction was mixed with 5 μL of ADP-Glo reagent, and then incubated at 21 °C. After 40 minutes. 10 μL of the Kinase Detection reagent was added and samples were incubated at 21 °C an additional 40 minutes. Luminescence from the converted ATP was then measured using a FilterMax F5 Multi-mode microplate reader (Molecular Devices).

### Imaging by immunofluorescence

MCF-7 and MCF-10A cells were seeded onto coverslips in 6-well (34.8 mm) plates at a density of 10,000 cells per well. Cells were allowed to adhere to coverslips for 24 hours and then treated with increasing doses of CB-5083 or a DMSO buffer control. 24 hours after drug treatment, cells were fixed for 10 minutes in 4% paraformaldehyde in PBS (137 mM NaCl, 2.7 mM KCl, 4.3 mM Na2HPO4, 1.47 mM KH2PO4, and pH adjusted to 7.4). Cells were then washed three times with PBS, incubated 1 hour with blocking buffer (5% FBS, 1% BSA, 0.2% Triton X-100 in PBS), and then incubated overnight with blocking buffer containing 1:500 anti-VCP antibodies (Fisher BDB612182). Cells were washed three times in PBS, incubated 1 hour with PBS containing 1:500 Alexa Fluor-488 (Fisher A-21202) secondary antibody, washed twice with PBS, and then mounted on slides using Vectashield containing DAPI (Vector). Z-stacked images of nuclei were taken with a step size of 1 μm using an Olympus FV10i LV laser scanning confocal microscope. Images were analyzed using FIJI. Z-stacked images were used to create a maximum Z projection and the pixel intensity was quantified. The relative fraction of nuclear p97 was represented as the total nuclear pixel intensity (average intensity x total nuclear area) divided by the total cellular pixel intensity (average intensity × total cellular area) normalized to the mean in buffer-treated cells. For cell clusters, nuclear and total intensities were determined in aggregate and used to determine the average abundance of each cell in the group.

### Cellular fractionation

MCF-7 and MCF-10A cells were obtained from ATCC and cultured according to their specifications. Two million cells were plated in 10 cm dishes for 24 hours, and then treated with fresh media containing the indicated concentration of CB-5083 or a DMSO buffer control. After 24 hours, treated cells were harvested with 0.5% trypsin-EDTA, washed with cold PBS and then fractionated using the Subcellular Protein Fractionation Kit (Thermo Scientific). Total samples of whole cell lysates were also prepared by resuspending pelleted cells in 2× SDS Sample Buffer. Proteins were resolved by SDS-PAGE and visualized by Western blotting with the indicated antibodies.

## Supplementary information

Supplementary Info
