# Supplementary material for: Uncoupling of p97 ATPase activity has a dominant negative effect on protein extraction
Source: Sci Rep. 2019 Jul 17;9:10329. doi: 10.1038/s41598-019-46949-4 (PMC6637110; doi:10.1038/s41598-019-46949-4)
Supplement: Supplementary file 1 — Supplementary Info [file 41598_2019_46949_MOESM1_ESM.pdf]

Supplemental Information

Uncoupling of p97 ATPase activity has a dominant negative effect on protein extraction

Halley B. Rycenga[1], Kelly B. Wolfe[1], Elizabeth S. Yeh[2], David T. Long[1]*

[1] Department of Biochemistry and Molecular Biology
Medical University of South Carolina, Charleston, SC 29425, USA
[2] Department of Pharmacology and Toxicology
Indiana University School of Medicine, Indianapolis, IN 46202, USA

* To whom correspondence should be addressed. Email: longdt@musc.edu

Supplemental Figures

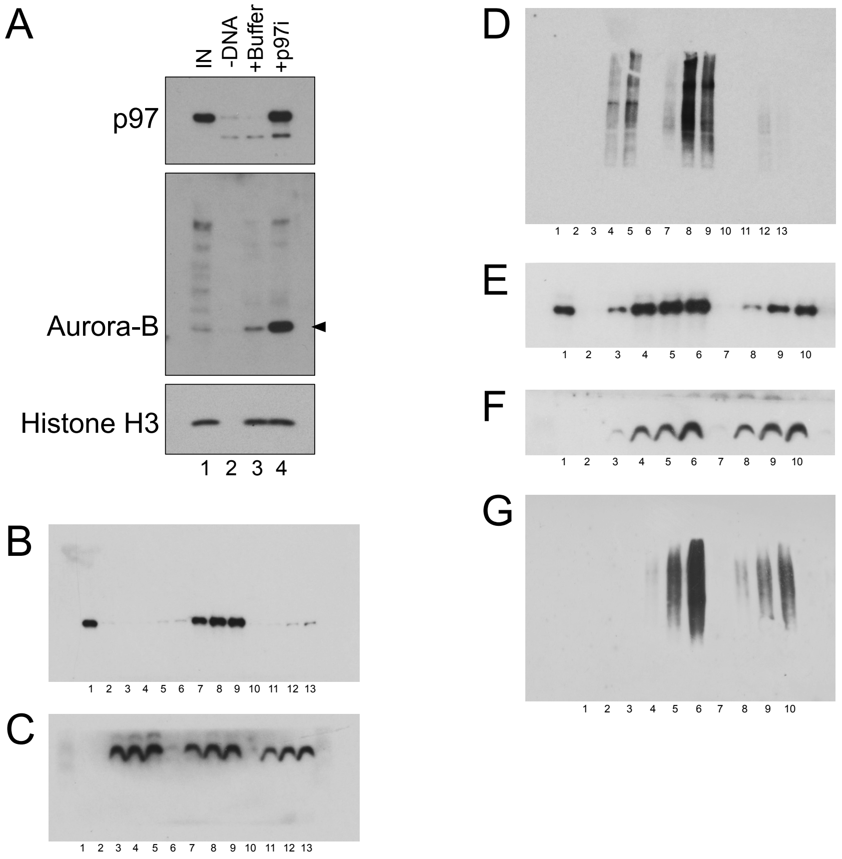

Figure S1. (**A**). pDNA was incubated in extract supplemented with buffer or CB-5083 (p97i) for 120 minutes. DNA-bound proteins were isolated by plasmid pull-down and visualized by Western blot with the indicated antibodies. Unmodified Aurora-B indicated by closed arrowhead. Full Western blots from Figure 1A: **(B)** p97, **(C)** Histone H3, and **(D)** Total ubiquitin. Full Western blots from Figure 1D: (**E**) p97, (**F**) Histone H3, and (**G**) Total Ubiquitin.

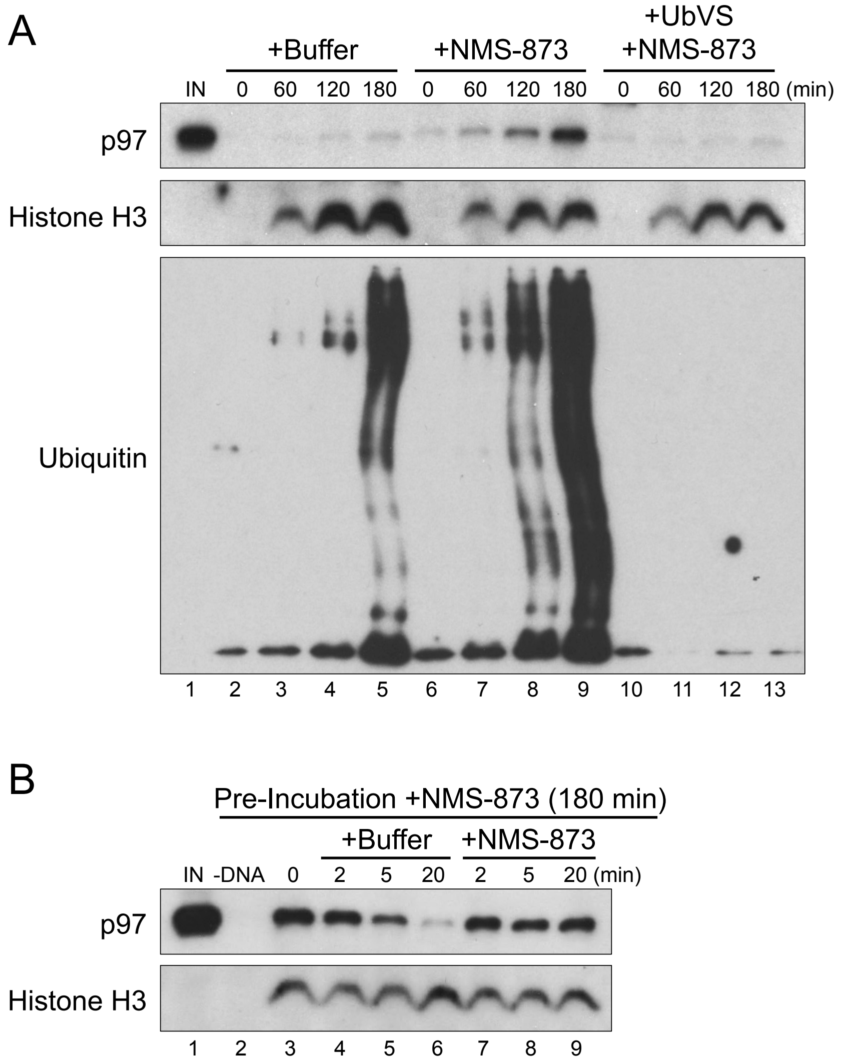
A
+Buffer
+NMS-873
+UbVS
+NMS-873
IN
0
60
120
180
0
60
120
180
0
60
120
180
(min)
p97
Histone H3
Ubiquitin
1
2
3
4
5
6
7
8
9
10
11
12
13
B
Pre-Incubation +NMS-873 (180 min)
+Buffer
+NMS-873
IN
-DNA
0
2
5
20
2
5
20
(min)
p97
Histone H3
1
2
3
4
5
6
7
8
9

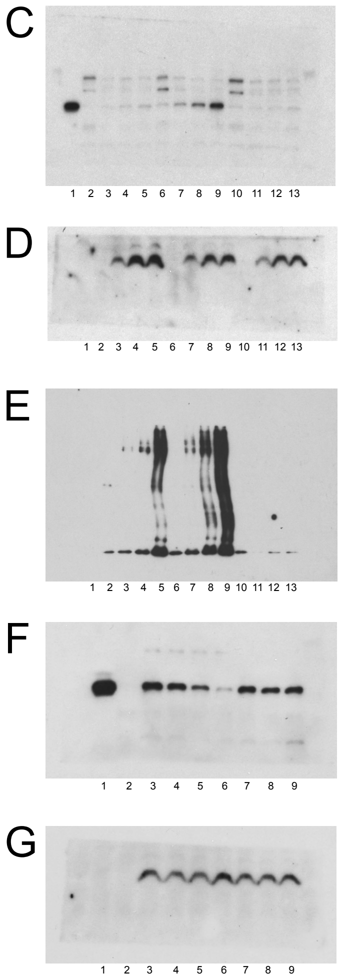

Figure S2. p97 inhibition with NMS-873. (A) pDNA was incubated in extract supplemented with Buffer, NMS-873, or NMS-873 and UbVS. DNA-bound proteins were isolated by plasmid pull-down at the indicated times and visualized by Western blot. **(B)** pDNA was incubated in extract supplemented with NMS-873. After 180 minutes, DNA was isolated by plasmid pull-down and then incubated in buffer with or without NMS-873. At various times, DNA-bound proteins were recovered and visualized by Western blot with the indicated antibodies. Full Western blot images are shown for *(A)* **(C-E)**, and *(B)* **(E-G)**.

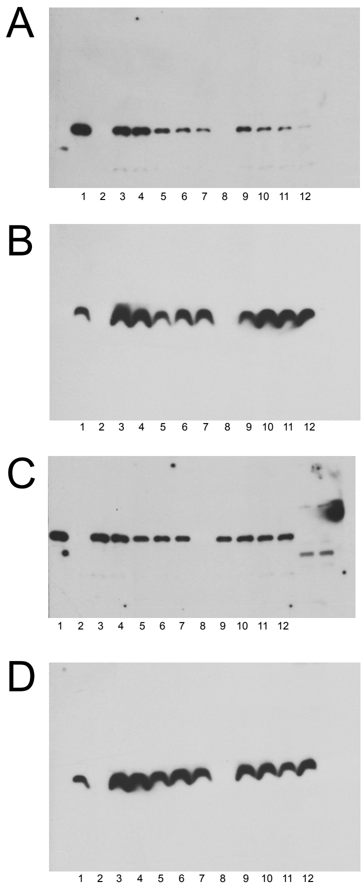

Figure S3. Full Western blots from Figure 2A: **(A)** p97, **(B)** Histone H3; and Figure 2B: **(C)** p97, **(D)** Histone H3.

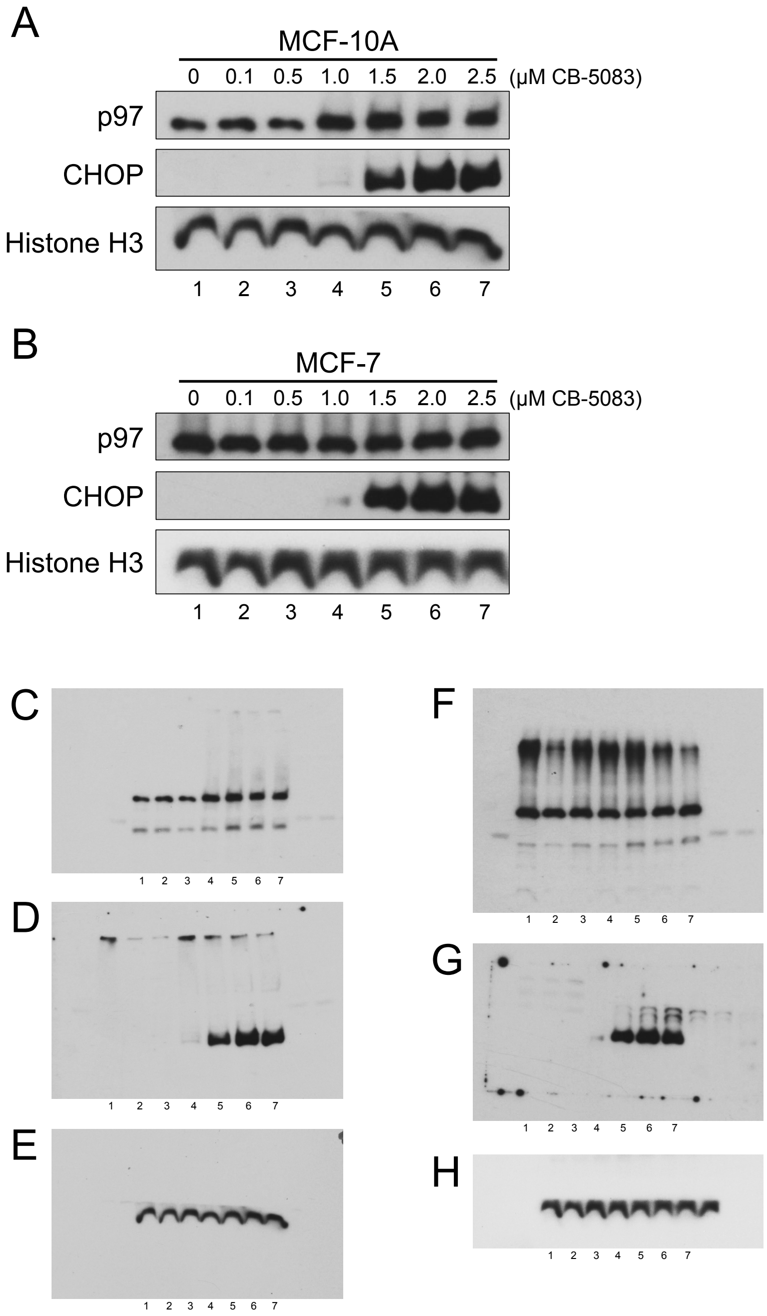

Figure S4. Protein levels in CB-5083-treated cells. (A) MCF-10A and **(B)** MCF-7 cells were treated with Buffer or the indicated concentration of CB-5083 for 24 hours. Total protein was analyzed from cell lysates by Western blot with the indicated antibodies. Full Western blot images are shown for *(A)* **(C-E)**, and *(B)* **(F-H)**.

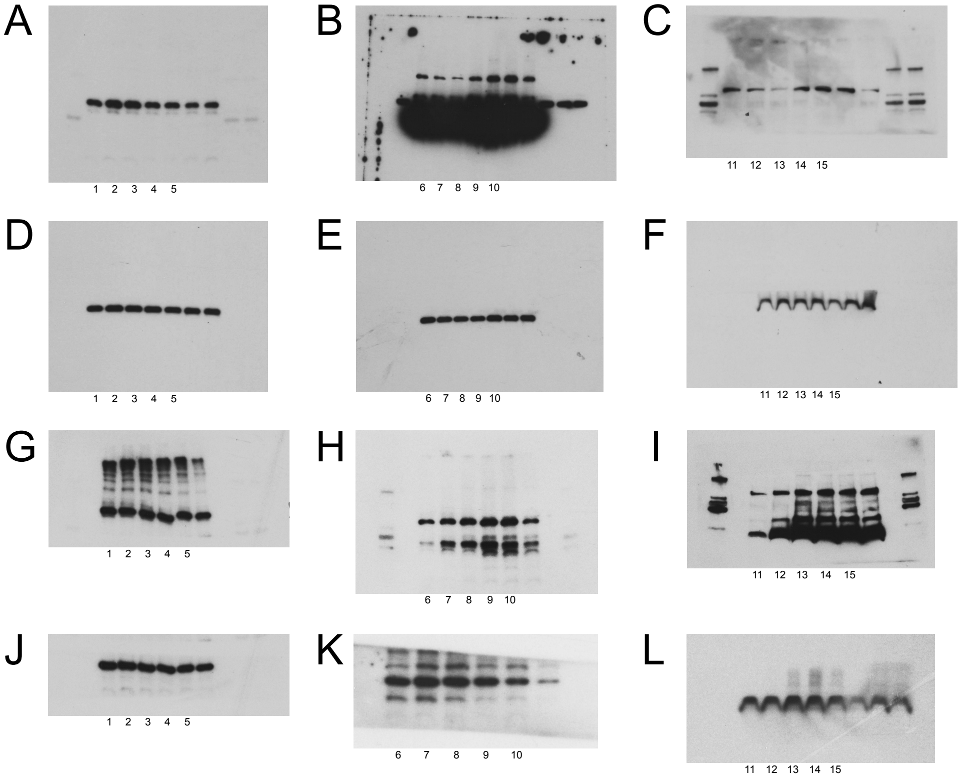

Figure S5. Full Western blots from Figure 4A: **(A)** Cytoplasmic p97, **(B)** Soluble nuclear p97 (with overexposed Actin below), **(C)** Chromatin-bound p97, **(D)** Cytoplasmic Actin, **(E)** Soluble nuclear Actin, **(F)** Chromatin-bound Histone H3; and Figure 4B: **(G)** Cytoplasmic p97, **(H)** Soluble nuclear p97, **(I)** Chromatin-bound p97, **(J)** Cytoplasmic Actin, **(K)** Soluble nuclear Actin, **(L)** Chromatin-bound Histone H3. Background bands in p97 blots are presumed to be non-specific or inactive degradation products and were not included in quantification.

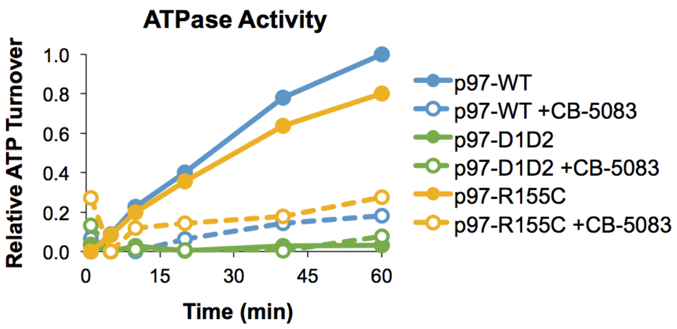

Figure S6. Recombinant p97 ATPase activity. Recombinant *Xenopus laevis* p97-WT, p97-D1D2, or p97-R155C was incubated in ELB with 100 mM ATP +/-CB-5083. At the indicated times, reactions were quenched and ADP formation was measured by luminescence. Values are normalized to ATP turnover in the p97-WT reaction.

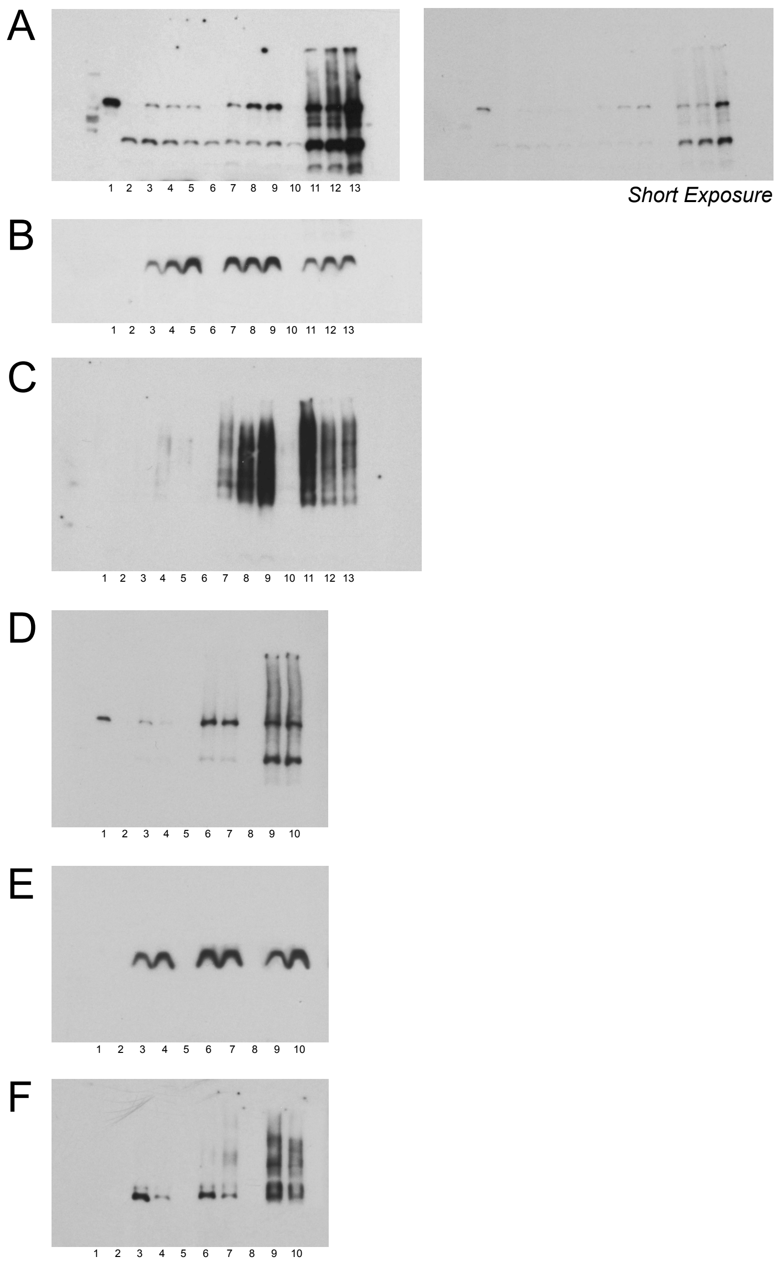

Figure S7. Full Western blots from Figure 5A: **(A)** p97, **(B)** Histone H3, **(C)** Total ubiquitin; and Figure 5D: **(D)** p97, **(E)** Histone H3, **(F)** Total ubiquitin.

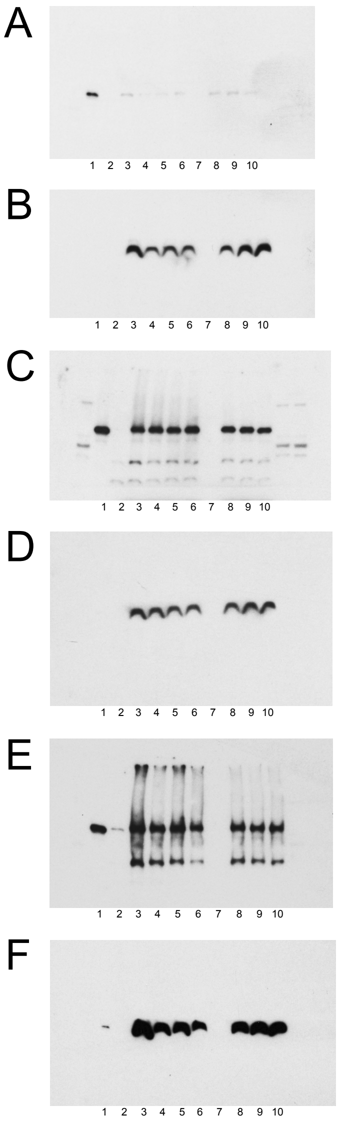

Figure S8. Full Western blots from Figure 6A: **(A)** p97, **(B)** Histone H3; Figure 6B: **(C)** p97, **(D)** Histone H3; and Figure 6C: **(E)** p97, **(F)** Histone H3.
